# Motor Resonance During Action Observation and Its Relevance to Virtual Clinical Consultations: Observational Study Using Transcranial Magnetic Stimulation

**DOI:** 10.2196/40652

**Published:** 2022-10-21

**Authors:** Urvakhsh Meherwan Mehta, Rakshathi Basavaraju, Abhishek Ramesh, Muralidharan Kesavan, Jagadisha Thirthalli

**Affiliations:** 1 Department of Psychiatry National Institute of Mental Health and Neurosciences Bengaluru India

**Keywords:** mirror neuron activity, virtual interactions, digital psychiatry, telepsychiatry, virtual mental health interventions, motor resonance

## Abstract

**Background:**

Virtual clinical interactions have increased tremendously since the onset of the COVID-19 pandemic. While they certainly have their advantages, there also exist potential limitations, for example, in establishing a therapeutic alliance, discussing complex clinical scenarios, etc. This may be due to possible disruptions in the accurate activation of the human mirror neuron system (MNS), a posited physiological template for effective social communication.

**Objective:**

This study aimed to compare motor resonance, a putative marker of MNS activity, estimated using transcranial magnetic stimulation (TMS) elicited while viewing virtual (video-based) and actual or real (enacted by a person) actions in healthy individuals. We hypothesized that motor resonance will be greater during real compared to virtual action observation.

**Methods:**

We compared motor resonance or motor-evoked potential (MEP) facilitation during the observation of virtual (presented via videos) and real (enacted in person) actions, relative to static image observation in healthy individuals using TMS. The MEP recordings were obtained by 2 single-pulse (neuronal membrane excitability–driven) TMS paradigms of different intensities and 2 paired-pulse (cortical gamma-aminobutyric acid-interneuron–driven) TMS paradigms.

**Results:**

This study comprised 64 participants. Using the repeated measures ANOVA, we observed a significant time effect for MEP facilitation from static to virtual and real observation states when recorded using 3 of the 4 TMS paradigms. Post hoc pairwise comparisons with Benjamini-Hochberg false discovery rate correction revealed significant MEP facilitation in both virtual and real observation states relative to static image observation; however, we also observed a significant time effect between the 2 action observation states (real > virtual) with 2 of the 4 TMS paradigms.

**Conclusions:**

Our results indicate that visual cues expressed via both virtual (video) or real (in person) modes elicit physiological responses within the putative MNS, but this effect is more pronounced for actions presented in person. This has relevance to the appropriate implementation of digital health solutions, especially those pertaining to mental health.

## Introduction

With the emergence of the COVID-19 pandemic, and the communication challenges in clinical scenarios thereof [1], we have witnessed an increasing worldwide reliance on digital media that support virtual clinical interactions [2]. This increase in virtual modes of communication has become ubiquitous, traversing many other spheres of daily living like work and education [3]. While there are definite advantages to using virtual communication tools in clinical settings [4], potential limitations do exist. Emerging research has identified challenges in establishing a therapeutic alliance in psychotherapy delivered via face-to-face videoconferencing modes [5]. Such challenges can potentially stem from an inaccurate perception and expression of thoughts and emotions while using virtual communication tools, which have been attributed to disruptions in the human mirror neuron system (MNS) during virtual or digital communications [6-8]. However, few studies have empirically examined the differences in putative MNS activity between virtual and real action observation scenarios. Activity within the human MNS can directly be measured only via intracranial depth electrodes [9], which comes with pragmatic challenges. Examining changes in cortical physiology to observe actions using alternative methods like transcranial magnetic stimulation (TMS), electroencephalography, functional neuroimaging, and others have therefore been more commonly used as indirect measures of putative MNS activity in humans [10].

## Methods

### Experiment

We present results from a TMS experiment in healthy volunteers that probed motor resonance or motor-evoked potential (MEP) facilitation during action observation relative to rest states, which is an indirect measurement of MNS activity in humans [11-14]. While there are no studies that definitively examine if motor resonance does indeed capture MNS activity, this method is one of the investigational approaches recommended for the study of possible MNS activity under the social processes of the US National Institute of Mental Health’s Research Domain Criteria [15]. Specifically, we compared MEP facilitation during the observation of virtual (presented via videos) and real (enacted in person) actions, relative to static image observation. The MEP recordings were obtained by 2 single-pulse (neuronal membrane excitability–driven) TMS paradigms of different intensities and 2 paired-pulse (cortical gamma-aminobutyric acid [GABA]-interneuron–driven) TMS paradigms [13,16]. We hypothesized that MEP facilitation will be greater during real compared to virtual action observation when elicited using all 4 TMS paradigms.

The data were obtained from 2 studies comparing motor resonance between patients with schizophrenia [17] or bipolar disorder [18] and healthy individuals. Data from only healthy individuals were used in this study. All participants provided written informed consent. Participants recruited as healthy individuals were screened for any current or past psychiatric morbidity using the Mini International Neuropsychiatric Interview screening checklist [19].

The TMS experiment was performed using a MagPro R30 system with MagOption (MagVenture); electromyography was obtained using a single-channel MEP monitor device mounted on the TMS device, and this data was analyzed using Signal-4 Software (Cambridge Electronic Devices). After localizing the motor hand area in the left hemisphere, we determined stimulus intensities to elicit 50-μV (resting motor threshold [RMT]) and 1-mV (SI_1mV_) amplitudes of MEP in at least 6 out of 10 trials. The mean (SD) for RMT and SI_1mV_ were 36.6 (SD 6.7) mV and 48.1 (SD 10.2) mV, respectively. Thereafter, we administered 10 pulses each of the 120% RMT, SI_1mV_, and short- and long-interval cortical inhibition (SICI and LICI, respectively) paradigms over the left motor cortex in a pseudorandom sequence at 5-second intervals while obtaining MEP recordings from the right first dorsal interosseus muscle. These recordings were obtained as the participants observed 3 states, presented in a random order across participants: (1) a static image of a hand and a lock and key, (2) a video of locking and unlocking actions with a key held in the right hand (virtual observation), and (3) the same action enacted by a volunteer (real observation). While SICI was measured at interstimulus intervals of 3 milliseconds between the subthreshold (80% RMT) and suprathreshold stimuli (SI_1mV_), LICI was measured at 100 milliseconds between 2 suprathreshold stimuli (SI_1mV_). SICI and LICI were expressed as a percentage of the ratio between the conditioned MEPs and the nonconditioned MEPs with SI_1mV_. MEP recordings with 120% RMT and SI_1mV_ were expressed in millivolts. In order to evaluate changes in MEP across the 3 experimental observation states (static, virtual, and real action observation), we performed a 1-way (within-subjects) repeated measures ANOVA. The omnibus tests for each TMS paradigm were 2-tailed, and results were regarded as significant at an α probability level (*P* value) of <.05. In addition, we performed post hoc pairwise comparisons with Benjamini-Hochberg false discovery rate correction to understand the statistical significance of MEP facilitation between pairs of experimental observation states.

### Ethics Approval

The National Institute of Mental Health and Neurosciences ethics committee approved the study protocols of the 2 studies [17,18] from which we derived the data (NIMHANS/71^ST^IEC/2010 and NIMH/DO/IEC (BEH.Sc.DIV)/2018).

## Results

This study comprised 64 participants (mean age 29.5, SD 8.5 years; females: n=31, 48%, males: n=33, 52%; mean years of education 13, SD 4.1 years).

The 1-way repeated measures ANOVA (Table 1) revealed a significant time effect for MEP facilitation from static to virtual and real observation states when recorded using the 120% RMT, SI_1mV_, and SICI paradigms, but not with LICI.

**Figure 1:**
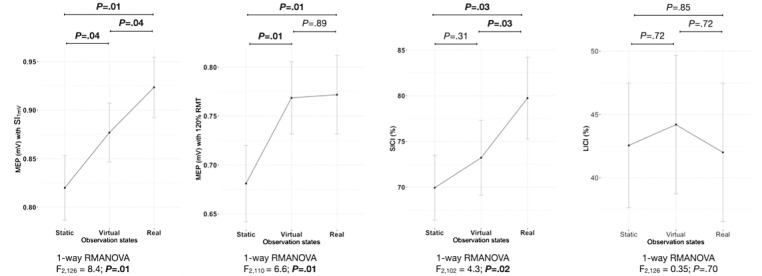
Motor evoked potentials during static, virtual, and real action observation conditions. Note: the data represent means and standard errors of the mean (error bars); *P* values for pair-wise comparisons (at the top of the plots) were obtained following post hoc Benjamini-Hochberg false discovery rate correction. LICI: long-interval cortical inhibition; MEP: motor-evoked potential; RMANOVA: repeated measures ANOVA; RMT: resting motor threshold; SI_1mV_: stimulus intensity to elicit 1 mV MEP; SICI: short-interval cortical inhibition.

Post hoc pairwise comparisons with Benjamini-Hochberg false discovery rate correction revealed significant MEP facilitation in both virtual (120% RMT and SI_1mV_) and real (120% RMT, SI_1mV_, and SICI) observation states relative to static image observation (Figure 1); moreover, there was also a significant time effect between the 2 observation states (real > virtual) for the SI_1mV_ and SICI paradigms (Figure 1).

**Table 1:** Motor-evoked potentials (in millivolts) with single- and paired-pulse stimulation paradigms during static and action observation experimental states.

TMS^a^ paradigm and observation states			Mean (SD)	*F* (*df*)^b^
**SI_1mV_^c^ (mV)**				8.4 (2, 126)^d^
	Static	0.82 (0.26)		
	Virtual action observation	0.87 (0.24)		
	Real action observation	0.92 (0.24)		
**120% RMT^e^ (mV)**				6.6 (2, 110)^d^
	Static	0.68 (0.31)		
	Virtual action observation	0.76 (0.29)		
	Real action observation	0.77 (0.32)		
**SICI^f,g^ (%)**				4.3 (2, 102)^d^
	Static	69.9 (28.4)		
	Virtual action observation	73.2 (32.7)		
	Real action observation	79.7 (35.8)		
**LICI^f,h^ (%)**				0.35 (2, 126)
	Static	42.6 (39.4)		
	Virtual action observation	44.2 (43.7)		
	Real action observation	42 (43.7)		

^a^TMS: transcranial magnetic stimulation.

^b^F statistic represents the time effect from a 1-way (within-subjects) repeated measures ANOVA.

^c^SI_1mV_: stimulus intensity to elicit 1 mV motor-evoked potential (MEP).

^d^*P*<.05.

^e^RMT: resting motor threshold.

^f^SICI and LICI were expressed as a percentage of the ratio between the conditioned and nonconditioned MEP with a stimulus intensity of SI_1mV_, that is, (conditioned MEP/nonconditioned MEP) × 100.

^g^SICI: short-interval cortical inhibition.

^h^LICI: long-interval cortical inhibition.

## Discussion

We observed motor resonance as evidenced by a significant time effect for MEP facilitation in both action observation states relative to static image observation using 3 out of 4 TMS paradigms. Even though MEP facilitation was observed in both real and virtual actions, the magnitude of this facilitation was significantly greater for real actions in 2 (SI_1mV_ and SICI) of the 3 paradigms. Together, these findings indicate that putative MNS activity was observed in response to both virtual and real action observation stimuli, but more so with the real action stimuli. It is perhaps reassuring, especially from a clinical scenario, that actions observed through virtual modes of communication elicit similar physiological responses as real enacted actions. This might partly explain how the quality of doctor-patient communication is broadly similar between video-based and face-to-face consultations [4,20]. However, virtual clinical encounters do demand more explicit forms of verbal communication [21] along with sufficient use of nonverbal gestures [22] than face-to-face consultations to ensure sufficient social information sharing. Our findings that motor resonance was greater in real (than virtual) actions may partly explain such limitations of video-based consultations and encourage the use of these technologies in less complex or less sensitive clinical situations and where there is already a trustful doctor-patient relationship in place [23].

Important caveats do exist in the interpretation of these results. We did not measure social information processing in real time as subjects observed actions. Further, the time effect was not significant for LICI; a similar lack of MEP facilitation and therefore motor resonance was noted with the LICI paradigm in our earlier study [17]. This might partly be due to the more robust inhibition of MEP observed in the GABA_B_ (metabotropic)-mediated LICI as opposed to the less pronounced inhibition of MEP in the GABA_A_ (ionotropic)-mediated SICI [24].

In summary, we provide preliminary evidence that visual cues expressed via both virtual (video) or real (in person) modes elicit physiological responses within the putative MNS, but this effect is more pronounced for actions presented in person. Future studies need to (1) replicate these observations in clinical contexts, using different approaches of eliciting putative MNS responses; (2) examine social cue perception and mental state attributions during virtual and in-person social interactions; and (3) examine the associations between cortical physiology (eg, putative MNS activity) and social cognition abilities across virtual and in-person social and clinical interactions. These findings will have relevance to the appropriate implementation of digital health solutions, especially those pertaining to mental health.

## Abbreviations

GABA: gamma-aminobutyric acid
LICI: long-interval cortical inhibition
MEP: motor-evoked potential
MNS: mirror neuron system
RMT: resting motor threshold
SICI: short-interval cortical inhibition
TMS: transcranial magnetic stimulation
